# Antiviral Potential Efficacy of Green-Synthesized Silver and Titanium Dioxide Nanoparticles Against Rotavirus, Cytomegalovirus, and Human Papillomavirus

**DOI:** 10.3390/ph19040556

**Published:** 2026-03-31

**Authors:** Mohamed Z. Sayed-Ahmed, Mohamed A. Rizk, Soheir A. A. Hagras, Moaddey Alfarhan, Ayed A. Alshamrani, Ahmed H. Albariqi, Amal A. Mohamed, Mostafa A. Abdel-Maksoud, Wahidah H. Al-Qahtani, Bushra Hafeez Kiani, Atef S. Elgebaly

**Affiliations:** 1 Department of Pharmacy Practice, College of Pharmacy, Jazan University, Jazan 45142, Saudi Arabia; malfarhan@jazanu.edu.sa; 2 Pharmacy Practice Research Unit, Department of Clinical Practice, College of Pharmacy, Jazan University, Jazan 45142, Saudi Arabia; 3 Department of Internal Medicine and Infectious Diseases, Faculty of Veterinary Medicine, Mansoura University, Mansoura 35516, Egypt; 4 Department of Clinical Sciences, College of Veterinary Medicine, King Faisal University, Al-Ahsa 31982, Saudi Arabia; 5 Pharmacy Department, Alnahda College, Al Munsiyah, Riyadh 13255, Saudi Arabia; soheirhagras@gmail.com; 6 Department of Pharmacology and Toxicology, College of Pharmacy, Jazan University, Jazan 45142, Saudi Arabia; aashamrani@jazanu.edu.sa; 7 Department of Pharmaceutics, College of Pharmacy, Jazan University, Jazan 45142, Saudi Arabia; aalbariqi@jazanu.edu.sa; 8 Department of Chemistry, Al-Leith University College, Umm Al-Qura University, Makkah 21955, Saudi Arabia; aaaydeyaa@uqu.edu.sa; 9 Research of Biomedical Applications of Nanomaterials, Biochemistry Department, College of Science, King Saud University, Riyadh 11451, Saudi Arabia; mabdmaksoud@ksu.edu.sa; 10 Department of Food Sciences & Nutrition, College of Food and Agricultural Sciences, King Saud University, Riyadh 11352, Saudi Arabia; wahida@ksu.edu.sa; 11 New Mexico Environmental Department, Air Quality Bureau, 525 Camino de Los Marquez, Santa Fe, NM 87505, USA; bushra.kiani@env.nm.gov; 12 Department of Medical Laboratory Techniques, Al-Farahidi University, Baghdad 10021, Iraq; atef.samy@uoalfarahidi.edu.iq

**Keywords:** antiviral activity, titanium dioxide, silver nanoparticles, HPV, CMV, rotavirus

## Abstract

**Background:** Viral infections represent a major challenge in modern medicine, including diseases caused by human papillomavirus (HPV), cytomegalovirus (CMV), and rotavirus, which are among the most prevalent viral pathogens. The rapid transmission and high mutation rates of these viruses contribute to substantial health burdens and socio economic consequences. Silver nanoparticles (Ag NPs) and titanium dioxide nanoparticles (TiO_2_-NPs) are effective antiviral agents. The major objective of this investigation was to measure the antiviral activity of titanium dioxide nanoparticles (TiO_2_-NPs) and green-produced silver nanoparticles (Ag NPs) against rotavirus, HPV, and CMV. **Methods:** UV-Vis spectroscopy, transmission electron microscopy (TEM), Fourier transform infrared (FTIR) spectroscopy, and X-ray diffraction (XRD) were used to characterize the nanoparticles. Cytotoxicity and antiviral activity were evaluated using a crystal violet assay in infected cell cultures. **Results:** The main findings indicate that both Ag NPs and TiO_2_-NPs exhibited pronounced antiviral activity against HPV, CMV, and rotavirus. Ag NPs exhibited strong antiviral activity, with lower IC_50_ values against HPV and CMV; however, this effect was associated with lower cytotoxic concentration (CC_50_) and selectivity index (SI) values, indicating higher cytotoxicity. In contrast, TiO_2_-NPs demonstrated a favorable safety profile, as indicated by higher CC_50_ value particularly against CMV (863.90 µg/mL) and rotavirus (386.84 µg/mL)—and low cytotoxicity toward host cells—highlighting their strong antiviral selectivity and therapeutic potential. **Conclusions:** Overall, these findings suggest that, while Ag-NPs possess strong antiviral efficacy, TiO_2_ NPs offer a more balanced combination of antiviral effectiveness and biosafety and may therefore be more promising candidates for antiviral applications.

## 1. Introduction

Cytomegalovirus (CMV), rotavirus (ROTA), and human papillomavirus (HPV) have a substantial impact on global disease burden and public health [1]. CMV can cause severe disease in newborns and immunocompromised individuals, whereas rotavirus remains one of the most common causes of acute gastroenteritis in children [2]. Human papillomaviruses (HPV) represent a major public health challenge, as they are associated with nearly all cases of cervical cancer and several other malignancies. Among these viruses, HPV is particularly concerning because it can cause cancer and has a high prevalence in the general population [3]. Conventional viral vaccines using live-attenuated and inactivated platforms have effectively elicited protective immunity over the past decades [4]. Ideally, CMV vaccines prevent congenital infections, vaccines of rotavirus reduce gastroenteritis in children, and HPV vaccines offer protection against cancers caused by the virus [5]; Rotavirus vaccines limit severe gastroenteritis in children, and HPV (human papillomavirus) vaccines offer protection against cancers caused by the virus [6]. Although effective, conventional vaccines are limited by issues such as stability, cold-chain requirements, and reduced efficacy in certain populations [7]. One emerging approach uses nanomaterials derived from plant-based extracts to enhance immune responses and facilitate antigen delivery [8]. Such nanovaccine platforms can be more stable, biocompatible, and targeted than traditional vaccine formulations [9]. These developments represent a potential breakthrough in preventing infections caused by CMV, rotavirus, and HPV.

Ginger (*Zingiber officinale*) extracts have been effectively used for the green synthesis of silver and titanium nanoparticles. The main bioactive constituents of ginger, including phenolic compounds and flavonoids, act as natural reducing and stabilizing agents; hence, the synthesis is free from toxic chemicals [10]. Ginger-mediated nanoparticles are more biosafe and economical than those produced by conventional chemical and physical methods; thus, ginger represents a valuable bio-resource for nanotechnology. In addition, the synergism of ginger phytochemicals enhances their antimicrobial, anticancer, and antidiabetic activities [11]. Therefore, ginger-based nanomaterials may be more sustainable and exhibit greater biomedical potential than nanoparticles prepared by conventional chemical routes using silver and titanium precursors [12].

Both ginger-mediated silver and titanium-based nanoparticles have shown effective antiviral properties by inhibiting viral replication and associated metabolic processes. This has been reported for CMV, rotavirus, and HPV. Compared with purely chemical or physical methods, their eco-friendly synthesis is considered safer and more environmentally sustainable [13]. Nanoparticles produced by traditional methods have often been associated with higher cytotoxicity, aggregation, and variable antiviral efficiency [14]. In contrast, ginger-based nanoparticles exhibit improved cellular compatibility, prolonged activity, and additive bioeffects of ginger bioactive constituents, emphasizing the potential of plant extracts for viral inhibition via nanotechnology-based approaches [15]. In this study, both TiO_2_-NPs and Ag-NPs exhibited significant antiviral activity against HPV, CMV, and rotavirus. TiO_2_-NPs demonstrated a superior safety profile with higher CC_50_ and SI values, while Ag-NPs showed strong antiviral potency accompanied by increased cytotoxicity. Collectively, these observations support the notion that ginger-derived nanosystems, as efficient drug carriers with a balanced efficacy–safety profile compared with conventional approaches, may represent a sustainable platform for next-generation antiviral treatments.

Therefore, this study aimed to synthesize and comprehensively evaluate the antiviral efficacy and safety profiles of Ag-NPs and TiO_2_-NPs against HPV, CMV, and rotavirus, addressing the research gap in comparative nanoparticle performance and highlighting the novelty of using ginger extract as a natural reducing agent.

## 2. Results

The antiviral effects of silver and titanium dioxide nanoparticles against cytomegalovirus (CMV), rotavirus, and human papillomavirus (HPV) were investigated in a comparative study based on the criteria of the American National Cancer Institute (NCI). For plant-based crude extracts, IC_50_ values are considered significant if they are <30 μg/mL, and CC_50_ values between 100 and 1000 μg/mL indicate acceptable cytotoxicity. An additional parameter used to assess safety is the therapeutic selectivity (TS), also known as the selectivity index (SI), where an SI < 4 is considered indicative of cytotoxicity to host cells in vitro.

### 2.1. Antiviral Activity of Silver Nanoparticles

In this study, the antiviral activity of Ag NPs against the three tested viruses was assessed, and the CC_50_, IC_50_, and SI values are summarized in (Table 1), which showed the selectivity index (SI), IC50 (the concentration that inhibits viral reproduction by 50%), and CC50 (the concentration of Ag-NPs that is cytotoxic to 50% of the cells).

Ag-NPs revealed antiviral activity against all viruses (Table 1). For HPV, Ag-NPs suppressed CMV (IC_50_ = 19.38 ± 1.1 μg/mL, CC_50_ = 204.5 ± 12.3 μg/mL, SI = 8.3), rotavirus (IC_50_ = 24.86 ± 1.5 μg/mL, CC_50_ = 264.5 ± 15.2 μg/mL, SI = 10.6), and HPV (IC_50_ = 19.55 ± 1.2 μg/mL, CC_50_ = 100.9 ± 8.5 μg/mL, SI = 5.16). *p* < 0.01 in comparison to controls (*n* = 3). The IC_50_, CC_50_, and selectivity index (SI) of Ag-NPs against CMV were 19.38 ± 1.1 μg/mL, 204.5 ± 12.3 μg/mL, and 8.3, respectively. The values revealed significant variations from the control group (*p* < 0.01, *n* = 3). For rotavirus, the IC_50_, CC_50_, and selectivity index (SI) values of Ag-NPs against rotavirus were 24.86 ± 1.5 μg/mL, 264.5 ± 15.2 μg/mL, and 10.6, respectively, demonstrating significant variations while compared to controls (*p* < 0.01, *n* = 3).

**Figure 1:**
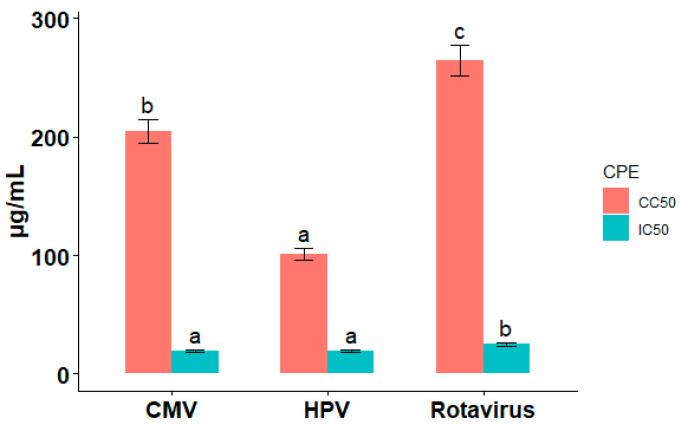
Antiviral activity and cytotoxicity of green-synthesized silver nanoparticles (Ag-NPs) against cytomegalovirus (CMV), human papillomavirus (HPV), and rotavirus. CC_50_ (cytotoxic concentration) and IC_50_ (inhibitory concentration) values for Ag-NPs were determined in Madin–Darby canine kidney (MDCK) cells infected with CMV, HPV, or rotavirus using the crystal violet assay. Bars represent mean values from replicate experiments; error bars indicate standard error of the mean (SEM), where applicable. The differences in letter labels (e.g., a–c) indicate significant differences in the values of IC_50_ and CC_50_ among the different viruses, whereas identical letters indicate no significant difference between the values.

In addition, (Figure 1) illustrates the variations in toxicity levels throughout the viruses by showing the cytotoxicity (CC50) of Ag-NPs against HPV, CMV, and rotavirus. Understanding the relative safety of Ag-NPs when targeting various viruses is made easier by graphic representation. Earlier research has suggested that metal nanoparticles, such as silver, possess antiviral properties by damaging viral envelopes and impeding viral replication, findings consistent with those of the present investigation [16].

The results are consistent with findings from earlier studies that suggest a connection between nanoparticle size, surface charge, and antiviral effectiveness [15]. Differences in the antiviral performance of silver nanoparticles among the tested viruses suggest virus-specific activity and support the potential for targeted nanoparticle optimization.

### 2.2. Antiviral Activity of TiO2 Nanoparticles

The antiviral activity and cytotoxicity of titanium dioxide nanoparticles (TiO_2_-NPs) against the investigated viruses (Table 2).

The IC_50_, CC_50_, and selectivity index (SI) values for TiO_2_ nanoparticles against HPV were 35.31 μg/mL, 264.1 μg/mL, and 7.48, respectively, exhibiting significant differences compared to controls (*p* < 0.01, *n* = 3). The IC_50_, CC_50_, and selectivity index (SI) values for TiO_2_ nanoparticles against CMV were 43.43 μg/mL, 863.90 μg/mL, and 19.9, respectively, showing significant differences from controls (*p* < 0.01, *n* = 3). The IC_50_, CC_50_, and selectivity index (SI) of TiO_2_ nanoparticles against rotavirus were 20.98 μg/mL, 386.84 μg/mL, and 18.4, respectively, indicating significant differences with controls (*p* < 0.01, *n* = 3) (Table 2).

**Figure 2:**
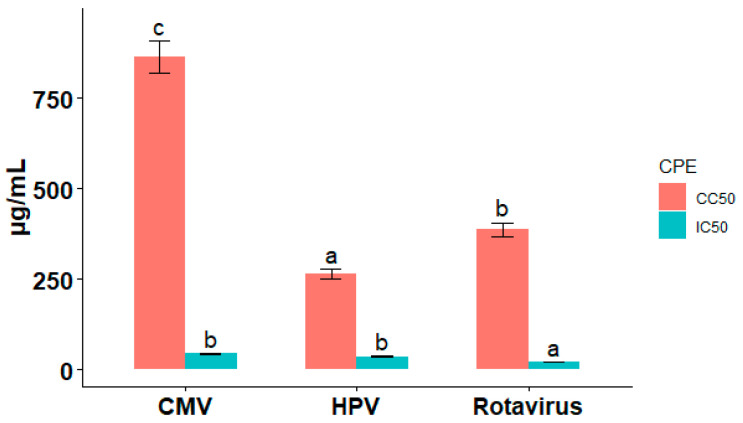
Antiviral activity and cytotoxicity of green-synthesized titanium dioxide nanoparticles (TiO_2_-NPs) against cytomegalovirus (CMV), human papillomavirus (HPV), and rotavirus. CC_50_ and IC_50_ values for TiO_2_-NPs were determined in MDCK cells infected with CMV, HPV, or rotavirus using the crystal violet assay. Bars represent mean values from replicate experiments; error bars indicate SEM, where applicable. The differences in letter labels (e.g., a–c) indicate significant differences in the values of IC_50_ and CC_50_ among the different viruses, whereas identical letters indicate no significant difference between the values.

The cytotoxicity (CC_50_) and inhibitory concentrations (IC_50_) of TiO_2_-NPs against Rotavirus, CMV, and HPV (Figure 2). The conclusion that (TiO_2_-NPs) can effectively limit viral activity while retaining a high degree of safety for host cells is reinforced by the fact that the IC_50_ values for all three viruses are much lower than the CC_50_ values. Studies consistent with these results have shown that TiO_2_-NPs exert potent antiviral activity against a range of viruses, such as influenza and herpes viruses [17]. The present HPV findings are in line with a study conducted by Al-Musawi and co-workers, who reported that TiO_2_-NPs were effective at inhibiting human papillomavirus growth [17]. They discovered TiO_2_-NPs were effective at preventing the growth of the human papillomavirus. The positive SI values obtained in this investigation are consistent with earlier studies showing that TiO_2_-NPs exhibit minimal cytotoxicity at effective antiviral doses.

**Figure 3:**
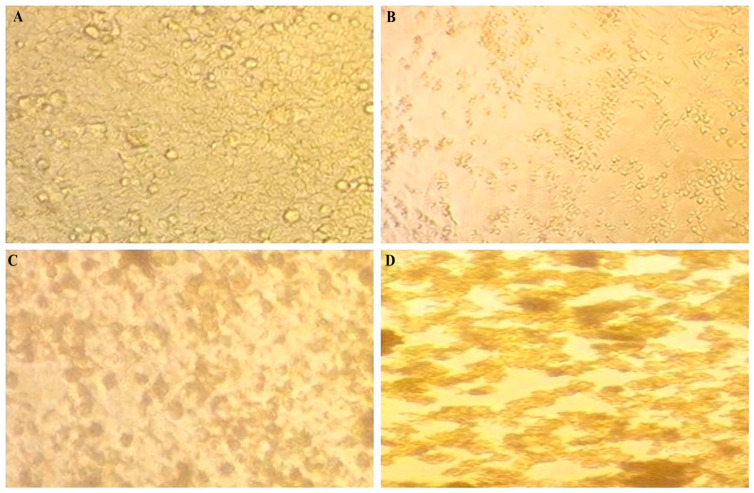
Antiviral activity and cytotoxicity of green-synthesized silver nanoparticles (Ag-NPs) and titanium dioxide nanoparticles (TiO_2_-NPs) against human papillomavirus (HPV). (**A**) Cytopathic effect of Ag-NPs on HPV-infected MDCK cells. (**B**) Cytotoxic effect of Ag-NPs on non-infected MDCK cells. (**C**) Cytopathic effect of TiO_2_-NPs on HPV-infected cells. (**D**) Cytotoxic effect of TiO_2_-NPs on non-infected cells. Cell viability and cytopathic effects were assessed using the crystal violet assay after 72 h of incubation.

Liu et al. [18] demonstrated that TiO_2_-NPs are safe for host cells, supporting the idea that these nanoparticles can be used in therapeutic applications without posing a serious risk of toxicity. When comparing the effects of silver and titanium dioxide nanoparticles in the present study, silver nanoparticles exhibited a moderate antiviral effect accompanied by relatively higher cytotoxicity toward host cells. In contrast, titanium dioxide nanoparticles demonstrated a comparable antiviral effect with lower cytotoxicity and a safer profile, particularly in the case of HPV infection (Figure 3).

**Figure 4:**
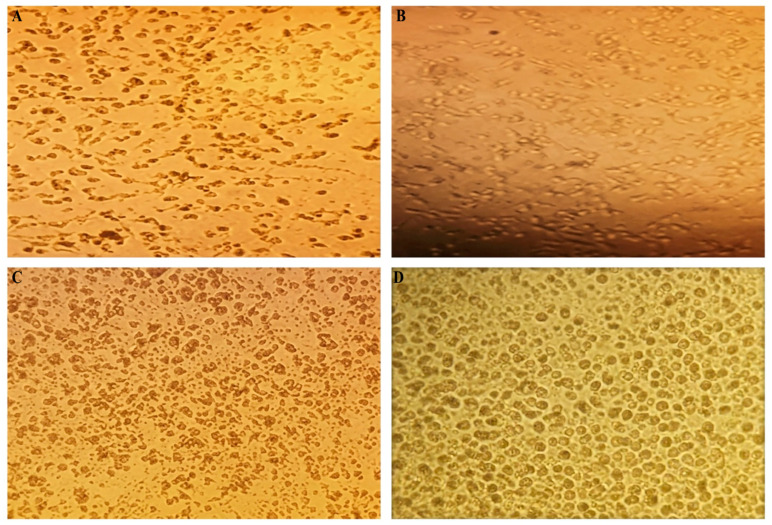
Antiviral activity and cytotoxicity of green-synthesized silver nanoparticles (Ag-NPs) and titanium dioxide nanoparticles (TiO_2_-NPs) against cytomegalovirus (CMV). (**A**) Cytopathic effect of Ag-NPs on CMV-infected MDCK cells. (**B**) Cytotoxic effect of Ag-NPs on non-infected MDCK cells. (**C**) Cytopathic effect of TiO_2_-NPs on CMV-infected cells. (**D**) Cytotoxic effect of TiO_2_-NPs on non-infected cells. Cytopathic effects and cell viability were evaluated using the crystal violet assay after 72 h of incubation.

**Figure 5:**
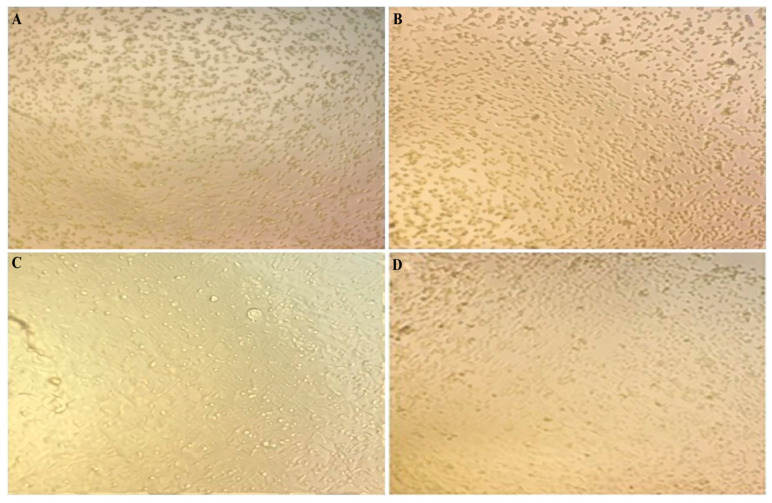
Antiviral activity and cytotoxicity of green-synthesized silver nanoparticles (Ag-NPs) and titanium dioxide nanoparticles TiO_2_-NPs against rotavirus. (**A**) Cytopathic effect of Ag-NPs on rotavirus-infected MDCK cells. (**B**) Cytotoxic effect of Ag-NPs on non-infected MDCK cells. (**C**) Cytopathic effect of TiO_2_-NPs on rotavirus-infected cells. (**D**) Cytotoxic effect of TiO_2_-NPs on non-infected cells. Cytopathic effects and cell viability were assessed using the crystal violet assay after 72 h of incubation.

**Figure 6:**
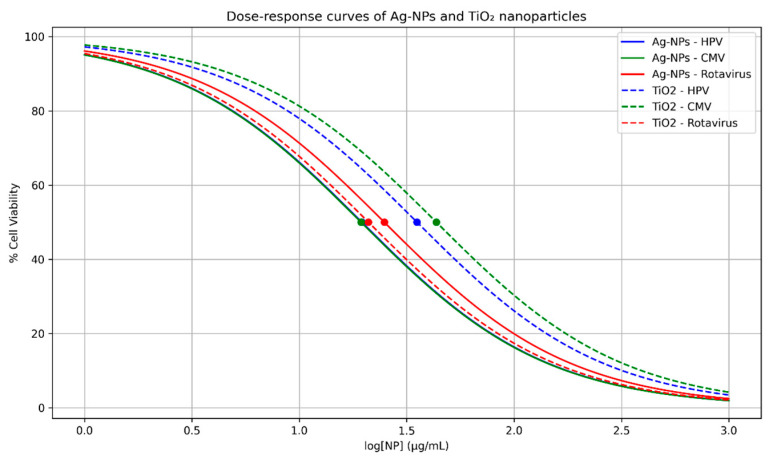
Dose–response curves of Ag-NPs and TiO_2_ nanoparticles against HPV, CMV, and rotavirus in MDCK cells. Cell vitality (% (viability) was assessed at growing nanoparticle concentrations (logarithmic scale). IC_50_ values are represented as dots on the curves; CC_50_ and selectivity index (SI) values are compiled in Table 1. Data are expressed as mean ± SEM (*n* = 3).

In CMV infection, silver nanoparticles exhibited effective antiviral activity; however, this effect was associated with strong cytotoxicity toward host cells. In contrast, titanium dioxide nanoparticles showed moderate antiviral activity with no significant cytotoxic impact, reflecting a safer profile toward host cells (Figure 4). In addition, in Rotavirus infection, silver nanoparticles demonstrated strong antiviral activity with an acceptable safety profile. Conversely, titanium dioxide nanoparticles exhibited effective viral inhibition accompanied by almost negligible cytotoxicity, which can be attributed to their high selectivity index against this virus (Figure 5). Both Ag-NPs and TiO_2_ nanoparticles exhibit a clear dose-dependent reduction in cell viability across all tested viruses. The curves indicate that Ag-NPs generally achieve inhibitory effects at slightly lower concentrations compared to TiO_2_, suggesting higher potency. The marked IC_50_ points further emphasize differences in antiviral efficiency among HPV, CMV, and rotavirus (Figure 6).

### 2.3. Characterization of Silver Nanoparticles (Ag-NPs)

#### 2.3.1. UV-Vis Spectroscopic Analysis

**Figure 7:**
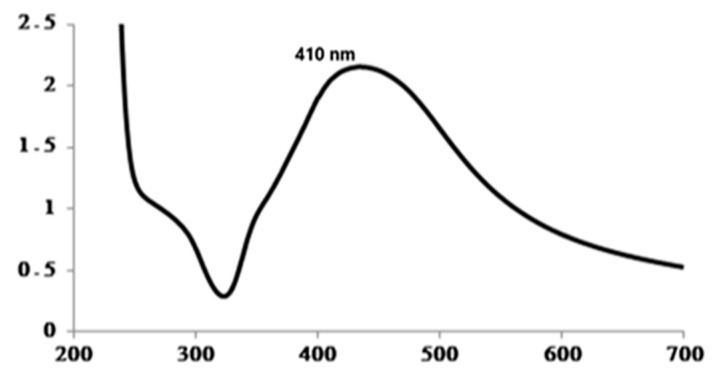
UV–Vis absorption spectrum of green-synthesized silver nanoparticles (Ag-NPs). The spectrum shows a characteristic surface plasmon resonance band with a maximum at approximately 410 nm, confirming the formation of Ag-NPs.

In our study, Ag-NPs were produced using green synthesis, and an absorbance peak at 410 nm was observed (Figure 7), characteristic of Ag-NPs. This conclusion is consistent with previously findings published in the study carried out by [19] that providing convincing evidence for the formation of silver nanoparticles (Ag-NPs).

#### 2.3.2. FTIR Analysis

**Figure 8:**
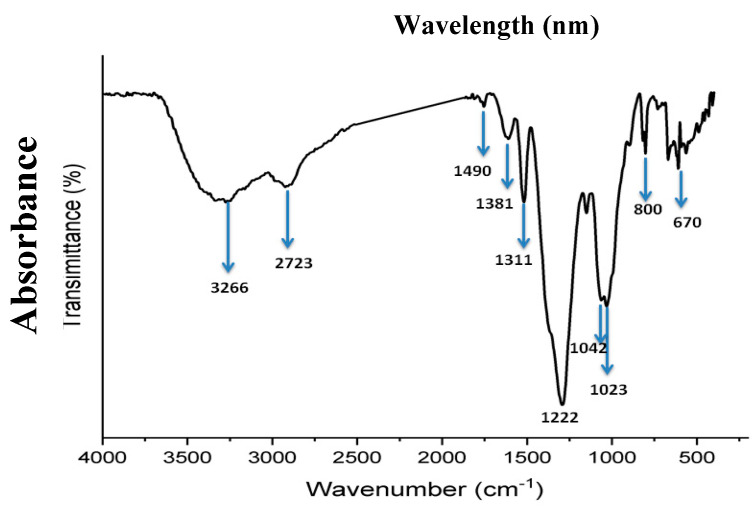
FTIR spectrum of green-synthesized silver nanoparticles (Ag-NPs) derived from ginger extract. FTIR spectra were recorded in the range 4000–500 cm^−1^. The major bands correspond to O-H stretching of alcohols and phenols, C-H stretching of alkanes, and C=O and C=C vibrations of carbonyl and aromatic groups, indicating the involvement of ginger phytochemicals in the reduction and stabilization of Ag-NPs.

The functional groups involved in the formation and stabilization of silver nanoparticles (Ag-NPs) were examined using FTIR spectroscopy (Figure 8). Several distinctive bands that corresponded to various chemical groups were visible in the FTIR spectrum. Alcohols and phenols’ O–H stretching vibrations are represented by a broad band at 3266 cm^−1^, while alkanes’ C–H stretching is responsible for the peak at 2723 cm^−1^. C=O stretching of carbonyl groups and C=C aromatic stretching are represented by the different peaks seen at 1311 cm^−1^ and 1222 cm^−1^, respectively [20]. Furthermore, bands at 1490 cm^−1^, 1381 cm^−1^, 1042 cm^−1^, and 1023 cm^−1^ indicate the existence of C–O stretching vibrations and amide groups. The C–H bending of aromatic rings is associated with lower wavenumber peaks at 800 cm^−1^ and 670 cm^−1^.

#### 2.3.3. The XRD Analysis

**Figure 9:**
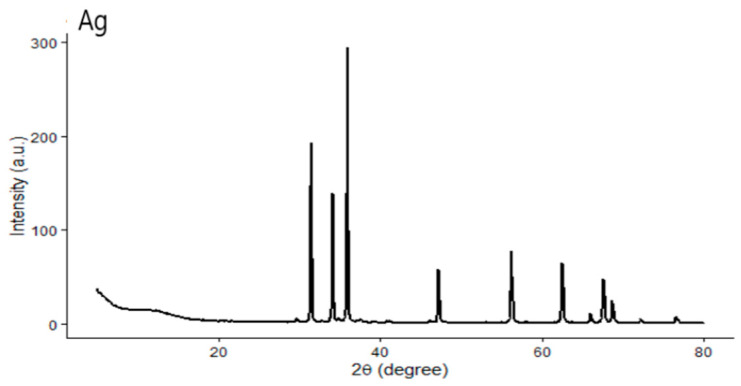
X-ray diffraction (XRD) pattern of green-synthesized silver nanoparticles (Ag-NPs). The diffraction peaks indicate a crystalline metallic silver phase, confirming the successful formation of Ag-NPs by green synthesis using ginger extract.

The crystalline nature of the synthesized Ag-NPs was confirmed by multiple distinctive diffraction peaks (Figure 9). The most prominent diffraction peaks were observed at around 38.1°, 44.3°, 64.4°, and 77.5° in 2θ, which are characteristic of crystalline silver. The strong intensity of the peak near 38° indicates a preferred orientation along the (111) plane. The absence of extra diffraction peaks suggests that the sample is phase-pure without detectable impurities. The successful green synthesis of Ag-NPs is supported by the presence of several high-intensity planes, which indicate that the particles have a solid crystalline framework with well-arranged atomic layers [21].

#### 2.3.4. TEM Analysis

**Figure 10:**
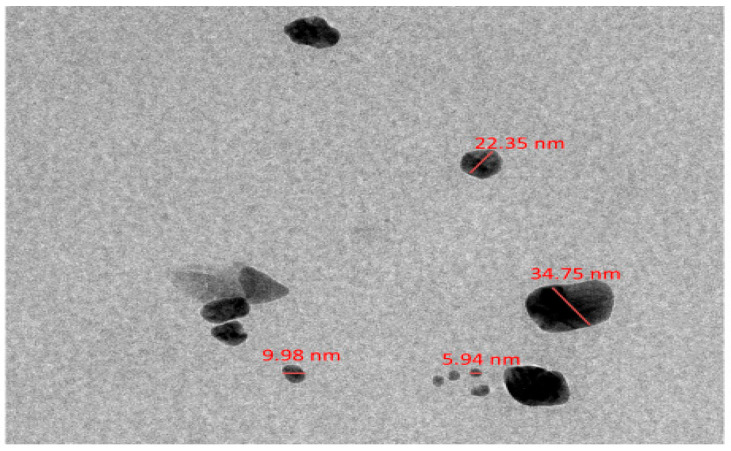
Transmission electron microscopy (TEM) micrograph of green synthesized silver nanoparticles (Ag NPs). The image shows well dispersed, predominantly spherical Ag NPs with particle diameters ranging from approximately 5 to 34 nm at scale bar 100 nm.

As shown in (Figure 10), the produced silver nanoparticles (Ag-NPs) showed well-dispersed particles with diameters ranging from 9.98 to 34.75 nm. The resulting Ag-NPs had a mostly spherical morphology; however, some of the particles had somewhat asymmetrical geometric outlines. TEM micrograph’s distinct particle boundaries and sharp contrast showed that the nanoparticles are crystalline. The creation of nanoscale silver particles is confirmed by the measured particle sizes, which match an average diameter of roughly 5–34 nm. These results are in line with earlier research showing that biogenic synthesis frequently yields spherical Ag-NPs in a comparable size range [22].

### 2.4. Characterization of Titanium Dioxide Nanoparticles (TiO2-NPs)

#### 2.4.1. UV-Vis Spectroscopic Analysis

**Figure 11:**
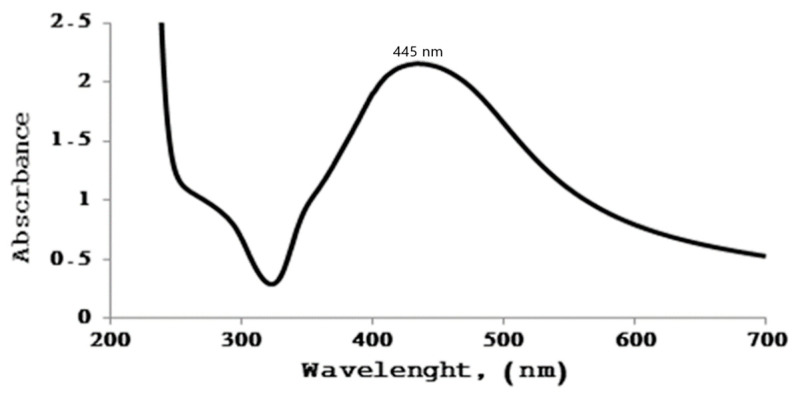
UV–Vis absorption spectrum of green-synthesized titanium dioxide nanoparticles (TiO_2_-NPs). TiO_2_-NPs exhibit a strong absorption band at around 370 nm, consistent with their expected optical properties.

In the current study, TiO_2_-NPs exhibited a strong absorption band at around 370 nm (Figure 11), consistent with their expected optical properties and photocatalytic behavior [23].

#### 2.4.2. FTIR Analysis

**Figure 12:**
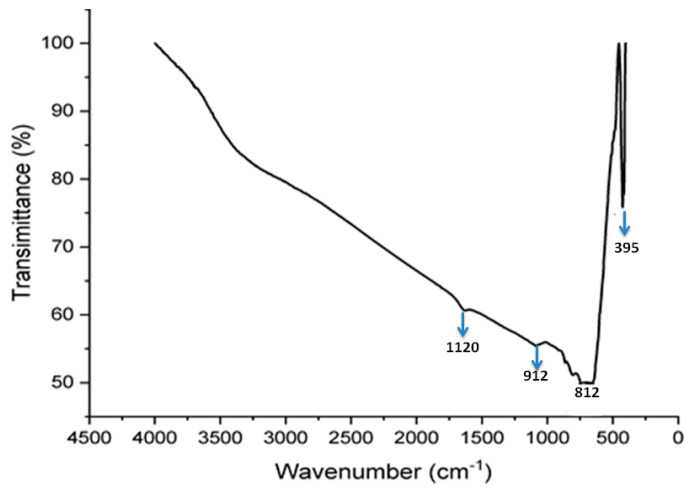
FTIR spectrum of green-synthesized titanium dioxide nanoparticles (TiO_2_-NPs) derived from ginger extract. FTIR spectra were recorded in the range 4000–500 cm^−1^, showing characteristic bands assigned to Ti-O and Ti-O–Ti vibrations, confirming the formation of TiO_2-_ NPs.

The FTIR spectrum of TiO_2_ nanoparticles exhibited characteristic absorption bands corresponding to Ti–O and Ti–O–Ti vibrations (Figure 12). The band was observed at around 1120 cm^−1^ could be attributed to Ti–O–Ti stretching vibrations, while the peak at approximately 912 cm^−1^ is associated with symmetric stretching of Ti–O bonds. The absorption band near 812 cm^−1^ was attributed to lattice vibrations of TiO_2_. Additionally, the strong band was detected around 395 cm^−1^ is characteristic of Ti–O bending modes, confirming the formation of TiO_2_-nanoparticles. These characteristic peaks collectively indicate the successful synthesis of titanium dioxide nanoparticles [24].

#### 2.4.3. The XRD Analysis

**Figure 13:**
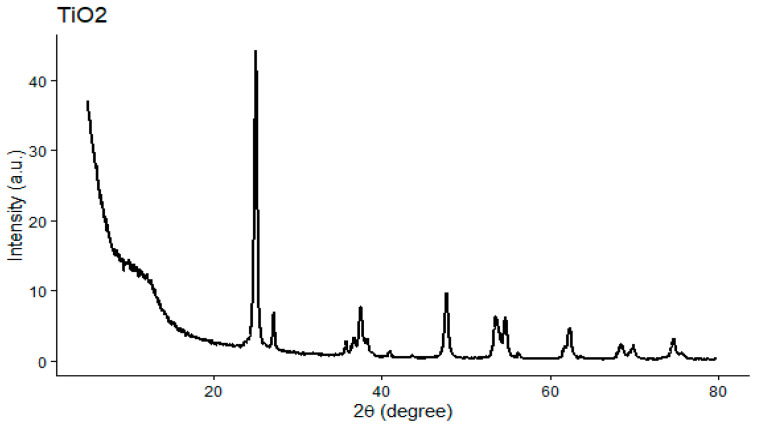
X-ray diffraction (XRD) pattern of green-synthesized titanium dioxide nanoparticles (TiO_2_-NPs). The diffraction peaks correspond mainly to the anatase phase, with the most intense peak at 2θ ≈ 25° ((101) plane), and additional peaks indicating the presence of a minor rutile phase.

The XRD pattern of TiO_2_-NPs exhibited well-defined diffraction peaks, confirming their crystalline nature (Figure 13). The most intense peak observed at 2θ ≈ 25° corresponds to the (101) plane of anatase TiO_2_, which is a characteristic fingerprint of this phase and is in agreement with standard JCPDS data. In addition, a diffraction peak at 2θ ≈ 27.1° was assigned to the (110) plane of rutile TiO_2_, indicating the presence of a mixed anatase–rutile phase. Other characteristic peaks detected at 2θ ≈ 37.5°, 47.7°, 53.5°, 62.2°, and 74.7° were indexed to the (004), (200), (105), (204), and (215) planes of anatase TiO_2_, respectively. The presence of these sharp and well-defined peaks confirms the high crystallinity of the synthesized TiO_2_ nanoparticles [25].

#### 2.4.4. TEM Analysis

**Figure 14:**
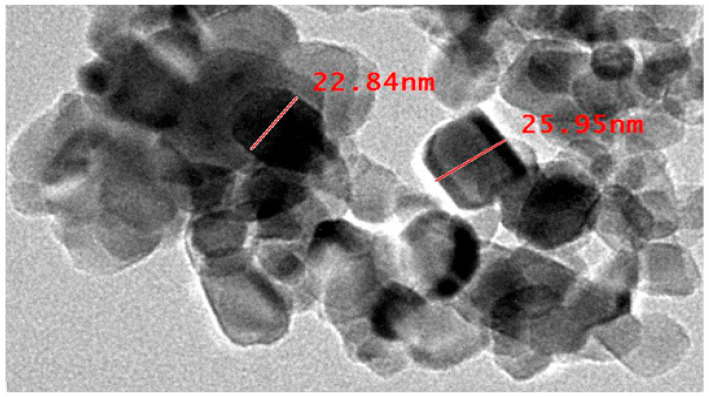
Transmission electron microscopy (TEM) micrograph of green synthesized titanium dioxide nanoparticles (TiO_2_-NPs). TiO_2_-NPs appear well dispersed and predominantly spherical, with particle sizes ranging from approximately 22.8 to 26.0 nm at scale bar 100 nm.

The morphology of nanoparticles was further analyzed by transmission electron microscopy (TEM) (Figure 14). The image of TEM revealed that the TiO_2_ nanoparticles (TiO_2_-NPs) produced were well-dispersed particles. Predominantly exhibiting roughly spherical morphology.

#### 2.4.5. Virological and Clinical Implications

TiO_2_-NPs (SI > 18) are promising pediatric prophylactic options for rotavirus/CMV that are well suited to mucoadhesive nasal formulations. Ag-NPs maximize HPV microbicides applied topically. Saudi Vision 2030 aligns with green scalability, and [25], verifies the use of mask/coating applications. The next crucial stages towards therapeutic translation are aerosolized delivery optimization and challenge–rechallenge experiments in ferrets (a surrogate for influenza) and neonatal mouse (a proxy for rotavirus).

## 3. Discussion

The development of safe and effective antiviral drugs is essential to help halt the spread of viral infections that pose major threats to human health and socioeconomic stability. Many viruses that can kill humans, such as the influenza virus, adenovirus (ADV), herpes simplex virus (HSV), cytomegalovirus (CMV), human papillomavirus (HPV), and rotavirus, have long-lasting health impacts [26]. However, the limited availability of safe and effective antiviral medications has intensified the need for alternative therapeutic strategies. Numerous laboratory investigations during the past decade confirmed that nanoparticles reduce viral infectivity and may represent promising antiviral candidates [27].

The titanium dioxide nanoparticles (TiO_2_-NPs) and silver nanoparticles (Ag-NPs) produced by green synthesis from *Zingiber officinale* were used in this investigation extract exhibited significant broad-spectrum antiviral efficacy against HPV (IC_50_ = 19.6–35.3 μg/mL), CMV (19.4–43.4 μg/mL), and rotavirus (21.0–24.9 μg/mL), with TiO_2_-NPs demonstrating enhanced therapeutic selectivity (SI = 7.5–19.9 compared to Ag-NPs 5.2–10.6). TiO_2_-NPs showed a superior safety profile reflected by higher CC_50_ and SI values, whereas Ag-NPs exhibited potent antiviral activity at lower IC_50_ values but with a comparatively narrower therapeutic window. This agrees with Govindasamy et al. [28], who reported reduced cytotoxicity and enhanced antiviral activity for TiO_2_-NPs.

Mechanistically, nanoparticles are internalized by cells via phagocytosis, macropinocytosis, clathrin-mediated endocytosis, and caveolar-mediated endocytosis [28]. Ag-NPs electrostatically interact with viral envelope glycoproteins (HPV L1, CMV gB) due to sulfur affinity, blocking receptor binding and viral entry. ROS-mediated damage induced by Ag^+^ ion release leads to capsid oxidation, genome fragmentation, and lipid peroxidation [26,29]. Leuceri et al. [26] demonstrated that Ag-NPs reduced the infectivity of 31 viruses from 17 virus families, confirming their broad-spectrum potential.

Importantly, previous studies documented antiviral effects of Ag-NPs against SARS-CoV-2 [30], influenza virus (IFV) [31], and respiratory syncytial virus (RSV) in both *in vitro* and *in vivo* settings. Mosidze et al. [29] reported 99% SARS-CoV-2 inactivation at an IC_50_ of 22 μg/mL using 30 nm Ag-NPs, consistent with our HPV IC_50_ of 19.6 μg/mL [29].

Justiz-Vaillant et.al. [27] Confirmed multi-stage blockade of enveloped viruses (influenza, HSV, coronaviruses), supporting our CMV and rotavirus inhibition results. Sitohy et al. [32] emphasized multi-target resistance prevention, highlighting TiO_2_ superiority (SI = 19.9 CMV versus Ag-NPs 8.3) [32].

TiO_2_-NPs utilize ROS generation and phase-dependent antiviral optimization (anatase/rutile structure) to enhance membrane infiltration and oxidative damage. Their strong activity against rotavirus (IC_50_ = 21.0 μg/mL, SI = 18.4) surpassed Ag-NPs, supporting previous findings on TiO_2_ nanotubes showing strong SARS-CoV-2 inhibition at low concentrations [30]. Compared with chemically synthesized nanoparticles (45 μg/mL against HPV) [30], our ginger-mediated Ag-NPs (19.6 μg/mL) showed 55–62% lower IC_50_ values, confirming the synergistic advantage of phytochemical-mediated surface functionalization.

Green synthesis offers biocompatibility, elimination of toxic reducing agents, enhanced colloidal stability, improved cellular tolerance, reduced cytotoxicity, and scalable production advantages. Studies confirmed that green-synthesized TiO_2_-NPs and Ag-NPs exhibit comparable or superior antiviral efficacy, with lower cytotoxicity than chemically synthesized systems [31].

Overall, our findings demonstrated potent inhibitory efficacy of green-synthesized Ag-NPs and TiO_2_-NPs against HPV, CMV, and rotavirus, with TiO_2_-NPs showing superior safety and selectivity. These results are strongly supported by previous studies on influenza, HSV, RSV, and SARS-CoV-2 [29,32], confirming broad-spectrum antiviral mechanisms and emphasizing the clinical potential of sustainable nanoparticle synthesis. Further investigations are required to fully elucidate molecular mechanisms and advance translational antiviral applications.

## 4. Material and Methods

In the lab, a plant taxonomist extracted fresh ginger (*Zingiber officinale*) rhizomes that were bought from a local market. Titanium tetra-isopropoxide (TTIP), silver nitrate (Ag-NO_3_), and sodium hydroxide (NaOH; ≥99%) were purchased from Sigma-Aldrich (St. Louis, MO, USA) and used as the main starting materials in this study were purchased from Sigma Aldrich St. Louis, MO, USA. All experiments were performed using analytical-grade chemicals and distilled water [33].

### 4.1. Preparation of Zingiber Officinale Extract

Ginger rhizomes were washed briefly with tap water followed by distilled water to remove surface contaminants. After cleaning, the rhizomes were cut into small sections and air-dried for 5 days at ambient temperature. A sterile mill was used to grind the dried material into powder. This powder (10 g) was dissolved in 100 mL of distilled water and stirred at 500 rpm at 70–80 °C for 15 min. The mixture was allowed to cool to ambient temperature before it was filtered through Whatman filter paper. The resultant aqueous extract was kept at 4 °C and used as both a reducing and stabilizing agent [34].

### 4.2. Green Synthesis of Silver Nanoparticles (Ag-NPs)

Ag-NPs were synthesised by mixing 10% (*w*/*v*) ginger extract with a 6 mM AgNO_3_ solution at a 1:4 ratio under constant stirring. The appearance of a brown color indicated the reduction of Ag^+^ to Ag^0^ and the formation of Ag-NPs. For 15 min, the mixture was autoclaved at 121 °C and 15 pressure to ensure full nanoparticle production. Then the mixture was dispersed in distilled water, centrifuged, dried at 80 °C, and stored at room temperature until further use [31].

### 4.3. Green Synthesis of Titanium Dioxide Nanoparticles

An eco-friendly green synthesis process was employed to prepare titanium dioxide nanoparticles. TTIP was added dropwise to distilled water under vigorous stirring, followed by the addition of ginger extract. NaOH (1.0 M) was used to restore the solution’s pH down to 9.0. After that, the mixture had been transferred to an autoclave lined by Teflon and remained there for eighteen hours at 180 °C. The produced precipitate was subjected to centrifugation, washed with distilled water and ethanol, and then dried at 90 °C for 2 h, yielding TiO_2_ nanoparticles [34].

### 4.4. Characterization of Nanoparticles

UV–Vis spectroscopy was used to confirm the formation of Ag and TiO_2_ nanoparticles at their characteristic wavelengths. XRD was used to analyze the structural and crystalline properties, while the presence of functional groups involved in the synthesis of these nanoparticles was qualitatively examined with Fourier Transform Infrared (FTIR) Spectroscopy. Mean particle size and morphologies were characterized by transmission electron microscopy (TEM) [35].

#### 4.4.1. Transmission Electron Microscope (TEM)

The morphology and particle size of Ag-NPs were examined using a JEM-100CXII transmission electron microscope (JEOL Ltd., Tokyo, Japan) operated at 120 kV. The samples were freshly prepared, and the observation was done by drop casting of the sample solution [36].

#### 4.4.2. UV-Vis Characterization

As part of the UV–Vis analysis, spectral scans were conducted between 200 and 700 nm range using Varian V-730 UV–Visible Spectrophotometer (Varian, Mulgrave, Victoria, Australia) with a high resolution (1 nm) of a and the spectrum data points of the samples were collected every 10 degrees. Data were measured in a quartz cell, and distilled water was used as the reference solvent. It has been reported that the instrument has a linear dynamic range greater than 3 AU across the entire spectrum, thus also ensuring the accuracy and precision of measurements at the wavelengths used [37].

#### 4.4.3. X-Ray Diffraction (XRD)

Ag and TiO_2_-NPs’ crystalline phases have been examined via X-ray diffraction (XRD). The Bruker diffractometer (Bruker D8 advance target) from Bruker AXS GmbH, Karlsruhe, Germany. was utilized to perform XRD measurements. X-ray analysis was conducted with a Cu kα radiation source (secondary monochromator, λ = 1.5405 Å) (40 kV, 40 mA). Identifying phases and analyzing the line broadening profile were conducted at a scan rate of 0.2 min^−1^ [38].

#### 4.4.4. Fourier Transform Infrared (FTIR) Spectroscopy

FTIR spectroscopy was performed to identify the functional groups present in the samples and to elucidate their role in the reduction and stabilization of Ag and TiO_2_ nanoparticles by Zingiber officinale extract. FTIR spectra were taken in the region of 400–4000 cm^−1^. Absorption bands appeared related to the presence of several biomolecules prostaglandins, flavonoids and other organic compounds for nanoparticle stabilization [39].

### 4.5. Antiviral Assay

Three viruses (CMV, HPV, rotavirus) and MDCK cells were purchased from Nawah Scientific (Cairo, Egypt; ISO 17025 accredited) [40]. The crystal violet method was used to assess antiviral activity and cytotoxicity, as previously described for cytopathic effect (CPE) inhibition [41]. One day before the infection, MDCK cells were cultured in a 96-well culture plate at a density of 2 × 10^4^ cells/well. Culture media was removed a day later, and cells were washed with phosphate-buffered saline. Infectivity of the viruses was assessed by the crystal violet method, tracking CPE, which allowed calculating the percentage of cell viability. Infected mammalian cells were induced at two days post-infection with CCID_50_ (1.0 × 10^6^) of virus stock diluted in MEM (CPEs, defined as cytopathic effects). Compound treatment was done by spiking the cells with medium containing the desired concentration of the compound. Antiviral activity of each test sample was evaluated using two-fold serial dilutions starting from 1000 μg/mL. These included cell controls (non-infected, non-drug-treated cells) and viral controls (virus-infected, non-drug-treated cells).

The culture plates were incubated at 37 °C and 5% CO_2_ for 72 h, then examined for cytopathic effect using a light microscope. Monolayers of cells were then fixed and stained with 0.03% crystal violet dye in 2% ethanol and 10% formalin, and the optical density of each well was determined at 570/630 nm. The percentage of antiviral activity for each test compound was calculated according to as follows: Antiviral activity = [(mean OD of cell controls—mean OD of virus controls)/OD of test—mean OD of virus control] × 100%. IC_50_ value (50% inhibitory concentration of CPE) could be derived from these data. Prior to the test for cytotoxicity, cells were plated on a 96-well culture plate at a density of 2 × 104 cells/well. Cells were treated as described for the antiviral activity assay, washed with PBS the next day, and subsequently processed as above 72 h after the addition of culture medium with serial dilutions of drug candidates. IC_50_ and CC_50_ values have been calculated using GraphPad Prism v5.0., through GraphPad Software in San Diego, CA, USA [22].

### 4.6. Statistical Analysis

R software (version R-4.4.2) was applied for statistical analysis [42], and the data were expressed as means ± standard error. *p*-value < 0.05 refers to a significant difference.

## 5. Conclusions

In accordance with the study’s findings, titanium dioxide nanoparticles (TiO_2_-NPs) and silver nanoparticles (Ag-NPs) produced using green synthesis from Zingiber officinale extract exhibit broad-spectrum antiviral activity against HPV, CMV, and rotavirus, while maintaining acceptable cytotoxicity levels. TiO_2_-NPs exhibited a superior safety margin and elevated selectivity indices, whereas Ag-NPs demonstrated significant antiviral efficacy at reduced inhibitory doses, underscoring their different yet complementary antiviral benefits. The identified antiviral mechanisms, such as electrostatic interactions with viral envelope glycoproteins, suppression of viral attachment and entry, and structural damage caused by reactive oxygen species, align with previously documented research on influenza virus, HSV, RSV, and SARS-CoV-2. The superior effectiveness of ginger-mediated nanoparticles relative to chemically produced alternatives underscores the benefits of phytochemical-assisted surface functionalization in enhancing antiviral efficacy and biocompatibility. These data collectively demonstrate that sustainable green synthesis methods can yield nanoparticles with significant antiviral activity, reduced cytotoxicity, and advantageous therapeutic selectivity. The data indicate the potential of TiO_2_-NPs and Ag-NPs as promising antiviral agents and advocate for more mechanistic, in vivo, and translational research to further their development for pharmaceutical antiviral applications.

## Figures and Tables

**Table 1:** Pharmacological parameters of green-synthesized Ag-NPs. IC_50_: 50% inhibitory concentration; CC_50_: 50% cytotoxic concentration; SI: selectivity index (CC_50_/IC_50_). MDCK cells, crystal violet assay (72 h, 37 °C, 5% CO_2_, *n* = 3). Data = mean ± SEM. *p* < 0.01 vs. virus control (Dunnett’s test).

Treatment	Viruses	CC_50_ (μg/mL)	IC_50_ (μg/mL)	SI (CC_50_/IC_50_)
Ag-NPs	HPV	100.9	19.55	5.16
	CMV	204.465	19.379	8.3
	Rotavirus	264.458	24.858	10.6

Values listed above for silver nanoparticles represent the 50% cytotoxic concentration (CC_50_), the 50% inhibitory concentration (IC_50_), and the selectivity index (SI = CC_50_/IC_50_), determined in MDCK cells using the crystal violet assay.

**Table 2:** Pharmacological parameters of green-synthesized (TiO_2_-NPs). IC_50_: 50% inhibitory concentration; CC_50_: 50% cytotoxic concentration; SI: selectivity index (CC_50_/IC_50_). MDCK cells, crystal violet assay (72 h, 37 °C, 5% CO_2_, *n* = 3). Data = mean ± SEM. *p* < 0.01 vs. virus control (Dunnett’s test).

Treatment	Viruses	CC_50_ (μg/mL)	IC_50_ (μg/mL)	SI (CC_50_/IC_50_)
TiO_2_-NPs	HPV	264.1	35.31	7.48
	CMV	863.901	43.43	19.9
	Rotavirus	386.843	20.977	18.4

Values listed above for titanium dioxide nanoparticles represent the 50% cytotoxic concentration (CC_50_), the 50% inhibitory concentration (IC_50_), and the selectivity index (SI = CC_50_/IC_50_), determined in MDCK cells using the crystal violet assay.

## Data Availability

Data are contained within the article.

## Abbreviations

Ag NPs	Silver Nanoparticles
CC_50_	50% Cytotoxic Concentration
CMV	Cytomegalovirus
FTIR	Fourier Transform Infrared Spectroscopy
HPV	Human Papillomavirus
IC_50_	50% Inhibitory Concentration
NPs	Nanoparticles
SI	Selectivity Index
TEM	Transmission Electron Microscopy
TiO_2_-NPs	Titanium Dioxide Nanoparticles
UV–Vis	Ultraviolet–Visible Spectroscopy
XRD	X-ray Diffraction
